# Supplementary material for: Extracranial Germ Cell Tumors in Children: Ten Years of Experience in Three Children’s Medical Centers in Shanghai
Source: Cancers (Basel). 2023 Nov 14;15(22):5412. doi: 10.3390/cancers15225412 (PMC10670163; doi:10.3390/cancers15225412)
Supplement: Supplementary file 1 [file cancers-15-05412-s001.zip › cancers-2607491-supplementary.pdf]

Supplementary Materials: Extracranial Germ Cell Tumors in Children: Ten Years of Experience in Three Children's Medical Centers in Shanghai

Shayi Jiang, Kuiran Dong, Kai Li, Jiangbin Liu, Xin Du, Can Huang, Yangyang Jiao, Yali Han, Jingwei Yang, Xuelian Liao, Yanhua Li, Ting Zhang, Shanshan Li, Zhibao Lv and Yijin Gao

Table S1. The survival of MGCTs with respect to various clinical factors.

Clinical characteristics	Case No.(%)	EFS			OS		
		EFS rate（95%CI）	χ^2	P	OS rate（95%CI）	χ^2	P
**Total**	298（100）	83.91(78.62, 7.99)			94.66(90.49, 7.03)		
**Gender**			0.0024	0.9607		0.1253	0.7234
Male	153(51.34)	84.47(77.14, 9.61)			94.88(89.54, 97.54)		
Female	145(48.66)	83.05(74.41, 8.99)			94.06(85.26, 97.67)		
Age			1.3715	0.2416		0.7464	0.3876
≥11 years	31(10.40)	76.49(54.53, 8.83)			90.48(67.00, 97.53)		
<11 years	267(89.60)	84.71(79.16, 8.89)			95.05(90.65, 97.40)		
**Primary site 1**			54.6044	<.0001		38.8531	<.0001
Mediastinum	11(3.69)	49.87(17.29, 5.90)			68.18(29.73, 88.61)		
Abdomen	16(5.37)	1.0000			1.0000		
Sacrococcygeal Region	57(19.13)	71.55(56.61, 2.12)			90.94(64.73, 97.95)		
Testis	105(35.23)	95.40(88.20, 8.25)			1.0000		
Ovary	63(21.14)	87.91(74.89, 4.42)			95.56(82.94, 98.90)		
Vagina	14(4.70)	1.0000			1.0000		
Pelvic cavity	20(6.71)	43.32(14.21, 9.87)			80.74(50.35, 93.55)		
Others	12(4.03)	74.07(39.07, 0.86)			83.33(48.17, 95.55)		
**Primary site 2**			20.5879	<.0001		9.0854	0.0026
Gonad	168(56.37)	92.66(86.76, 5.99)			98.49(94.04, 99.63)		
Extragonadal	130(43.62)	72.37(62.46, 0.08)			88.83(78.51, 94.37)		
**Primary site 3**			23.7765	<.0001		24.6744	<.0001
Mediastinum	9(3.02)	40.00(9.81, 69.65)			63.49(23.82, 86.60)		
Non-thorax	289(96.98)	85.30(80.03, 9.27)			95.74(91.63, 97.85)		
**Metastasis***			12.6924	0.0129		5.4206	0.2468
Non-metastasis	214(71.81)	87.88(81.90, 1.99)			96.58(92.49, 98.46)		
Metastasis but non pulmonary	12(4.03)	90.91(50.81, 8.67)			90.91(50.81, 98.67)		
Pulmonary Metastasis	29(9.73)	65.42(43.61, 0.49)			95.24(70.72, 99.32)		
Pulmonary and others metastasis	14(4.70)	69.84(37.79,7.60)			92.86(59.08, 98.96)		
**Tumor resection extent**			65.4451	<.0001		30.0533	<.0001
Complete resection	252(84.56)	89.79(84.68, 93.26)			97.00(92.62, 98.80)		
Partial resection	33(11.07)	40.22(21.06, 58.71)			76.30(52.85, 89.16)		
Unoperated	9(3.02)	74.07(28.92, 93.00)			88.89(43.30, 98.36)		
**Pathology**			17.7229	0.0005		3.0071	0.3905
Yolk sac tumor	183(61.41)	88.34(81.98, 92.55)			95.70(90.19, 98.15)		
Malignant mixed germ cell tumor	81(27.18)	69.47(56.49, 79.26)			91.80(81.10, 96.56)		
Sex cord stromal tumors	17(5.70)	82.05(44.42, 95.29)			88.89(43.30, 98.36)		
Others	17(5.70)	1.0000			1.0000		

**AFP1**			10.5842	0.0142		6.2758	0.0989
S0	38/(16.31)	89.72(71.20, 96.60)			95.83(73.92, 99.40)		
S1	46/(2.58)	97.14(81.40, 99.59)			1.0000		
S2	66/(28.33)	78.67(64.32, 87.77)			96.39(86.32, 99.09)		
S3	91/(39.06)	73.04(61.49, 81.64)			86.31(71.13, 93.83)		
**AFP2**			7.1259	0.0076		5.7929	0.0161
≥10000	92(30.87)	73.04(61.49, 81.64)			86.07(70.33, 93.81)		
<10000	140(46.98)	86.35(78.14, 91.64)			97.24(91.60, 99.11)		
**Stage**			27.5495	<.0001		10.0411	0.0182
I	113(37.92)	95.40(88.19, 98.25)			1.0000		
II	36(12.08)	94.28(78.98, 98.54)			94.28(78.98, 98.54)		
III	83(27.85)	76.44(63.29, 85.41)			93.39(82.94, 97.53)		
IV	66(22.15)	67.30(53.34, 77.91)			84.58(64.49, 93.81)		
**risk group**			21.3758	<.0001		7.6127	0.0222
Low risk	108(36.24)	95.39(88.17, 98.25)			1.0000		
Intermediate risk	84(28.19)	85.91(75.25, 92.21)			91.46(81.84, 96.10)		
High-risk	106(35.57)	70.39(58.91, 79.22)			89.97(75.12, 96.17)		
**Neoadjuvant chemotherapy**			1.3434	0.2464		0.0018	0.9666
Yes	146(48.99)	81.80(74.02, 87.44)			95.17(89.52, 97.81)		
No	134(44.97)	85.96(77.13, 91.56)			94.12(85.63, 97.66)		
**Chemotherapy regimen**			15.4377	0.0015		1.3222	0.7239
BEP	21(7.05)	94.12(65.02, 99.15)			94.12(65.02, 99.15)		
JEB	102(34.23)	71.48(58.11, 81.25)			94.96(86.91, 98.11)		
PEB	22(7.38)	1.0000			1.0000		
Others	102(34.23)	88.50(80.17, 93.46)			95.00(86.64, 98.18)		

* 28 cases with local tumor infiltration were excluded.
